# Secular trends in age at menarche among Han Chinese females in Shanghai: a large-scale community-based study

**DOI:** 10.1186/s12905-025-04064-9

**Published:** 2025-11-03

**Authors:** Xiaoli Xu, Genming Zhao, Xing Liu, Na Wang, Xiaohua Liu, Yonggen Jiang, Qian Peng, Jianhua Shi, Yuping Cheng, Mengru He, Dandan He, Huilin Xu

**Affiliations:** 1 Minhang District Center for Disease Control and Prevention (Minhang District Institute of Health Supervision), Shanghai, 201101 China; 2 School of Public Health, Fudan University, Shanghai, 200032 China; 3 Songjiang District Center for Disease Control and Prevention (Songjiang District Institute of Health Supervision), Shanghai, 201600 China; 4 Jiading District Center for Disease Control and Prevention (Jiading District Institute of Health Supervision), Shanghai, 201800 China; 5 Xuhui District Center for Disease Control and Prevention (Xuhui District Institute of Health Supervision), Shanghai, 200237 China

**Keywords:** Age at menarche, Secular trend, Overweight/obesity, Shanghai

## Abstract

**Background:**

Menarche, marking the onset of female reproductive potential. Understanding the secular trends in age at menarche (AAM) is crucial for public health, as earlier menarche is associated with adverse health outcomes in adulthood. This study sought to explore the long-term trends of AAM among Han Chinese females in Shanghai, China, and to explore its correlation with overweight/obesity in adulthood.

**Methods:**

We utilized baseline data from the Shanghai Suburban Adult Cohort and Biobank (SSACB), a large-scale community-based study. A total of 38,396 Han females born between 1942 and 1995 were included. Participants were divided into 11 birth-year cohorts and five percentile groups (10th, 25th, 50th, 75th, and 90th) based on AAM. The overweight/obesity status was defined using BMI criteria. Quadratic and quantile regression analyses were conducted to evaluate the time trends of AAM.

**Results:**

The mean AAM decreased from 17.45 ± 2.50 years in females born in 1942 to 14.20 ± 1.33 years in those born in 1995, with an average decline of 0.68 years per decade (*p* < 0.001). This trend was consistent across all percentile groups, with the most significant decline observed in the 90th percentile group. Females who experienced a greater decline in AAM (a decrease of 0.076 years per year) had a higher prevalence of overweight/obesity (OO) — compared to females with a smaller decline in AAM (a decrease of 0.064 years per year), who were currently non-overweight/obese (NOO).

**Conclusions:**

This study highlights a secular trend towards earlier menarche among Han Chinese females in Shanghai over the past half-century. Women with earlier menarche are more likely to be overweight or obese in adulthood, suggesting that earlier menarche may increase the risk of obesity in adulthood, and attention should be paid to early weight control intervention. Our findings provide valuable insights for public health policies aimed at improving Han women’s health in highly urbanized regions.

**Supplementary Information:**

The online version contains supplementary material available at 10.1186/s12905-025-04064-9.

## Introduction

Adolescence is a pivotal phase in the human life cycle [1]– [2], during which menarche, a critical milestone in female pubertal development, marks the initiation of female reproductive potential [3]. The age at menarche (AAM) is intricately modulated by genetic, environmental, and endocrine factors [4–10], exerting significant influence on long-term female health outcomes [11]. Empirical evidence has demonstrated that reproductive factors, including menarche, are related to a series of adverse health outcomes in women’s adulthood [12–21], among which women with early menarche are more likely to have obesity, diabetes, metabolic syndrome, some cancers, breast tumor and depressive disorders [17–21].

In females with early menarche (age < 12), premature activation of estrogen may disrupt lipid metabolism and insulin sensitivity, leading to abnormal synergistic effects between growth hormone and sex hormones, thereby triggering metabolic disorders in adulthood [17, 18]. Meanwhile, long-term estrogen stimulation promotes breast cell proliferation, increases the probability of DNA replication errors, and is accompanied by abnormal breast differentiation, making cells more sensitive to carcinogenic factors [19, 20]. In terms of mental health, premature fluctuations in sex hormones may affect prefrontal cortex development, leading to emotional regulation disorders. The mismatch between physical development and cognitive abilities may increase the risk of mental health issues [16, 21, 22].

Globally, a secular trend towards earlier menarche has been observed across a multitude of countries [23–27]. However, in some Western countries, the rate of decline has recently abated or plateaued [28]. Consistent with the global trend, a comprehensive longitudinal study evaluated the downward trend of AAM among 173,535 Han girls aged 9–18 in China over a 30-year period [25].

Although there have been reports on the long-term trends of AAM in different regions of China [29–34], Shanghai, as one of the most representative highly urbanized areas in eastern China, has only previously examined the changes in AAM among 5,207 women in Shanghai and surrounding areas [35]. Thus, there is a lack of large-scale sample data on the long-term trends of AAM among Han women in Shanghai. It also remains uncertain whether this trend varies by menarcheal age group percentile. However, research on the trend of AAM in Shanghai will have high reference value for highly urbanized areas and Han populations.

Globally, overweight and obesity have become a major epidemic among adult females. From 1990 to 2021, the global prevalence of obesity in females increased by 104.9% (from 10.2% to 20.8%), with approximately 1.11 billion adult females affected in 2021 [36]. Although China had the largest population of adults with overweight and obesity worldwide (reaching 402 million in 2021), the age-standardized prevalence of obesity among Chinese females remained relatively low (10.8%, 95% uncertainty interval 10.5–11.0). In contrast, the prevalence of obesity among females in high-income Western countries was significantly higher: for example, the prevalence among females in the USA reached 45.6% (95% uncertainty interval 43.7–47.5) in 2021 [36]. Future projections indicate that the prevalence of overweight and obesity among females in East Asia, including China, will increase more substantially, with an estimated rise of 57.9% (95% uncertainty interval 13.8–74.1) between 2021 and 2050, far exceeding the steady and slow growth in Western countries [36].

Therefore, this study leverages the Shanghai Suburban Adult Cohort and Biobank (SSACB) [37], a community-based resource, to fill the gap in large-scale sample data on AAM trends among Han women in Shanghai. Using data spanning over half a century, this study explored the trend of menarche age in different percentile groups and its correlation with overweight/obesity in adulthood, providing a practical basis for health administrative departments to adjust women’s health policies and protect women’s health.

## Materials and methods

### Study population

This study leveraged baseline questionnaire data and standardized physical examination results from the SSACB. Comprehensive details regarding SSACB are documented in a prior publication [37]. In brief, four districts in Shanghai (Songjiang, Minhang, Xuhui, and Jiading) were initially selected based on demographic and economic criteria. A multi-stage stratified random sampling approach was then employed to randomly select approximately one-third of the communities within each district as study sites. The study recruited individuals who had resided in Shanghai for at least five years and were aged between 20 and 74 years.

From 2016 to 2019, a total of 69,116 individuals participated in the baseline phase of this prospective cohort study. All participants completed a structured baseline questionnaire, which captured sociodemographic characteristics, including gender, age, birth history, and educational attainment. Concurrently, participants underwent standardized physical examinations conducted by trained technicians and clinicians, yielding data on physical examination results, including height and weight measurements.

Initially, we excluded 27,986 males, 1,028 individuals who did not report their AAM, and 1,497 individuals with implausible AAMs (Previous studies have not recorded the menarche age of infants aged 0–3 years [22–34]). Subsequently, 118 non-Han females and individuals born after 1995 (totaling 91 individuals, with fewer than 50 individuals per birth year) were excluded. Ultimately, 38,396 Han Chinese females from Shanghai were included in the study.

**Fig. 1:**
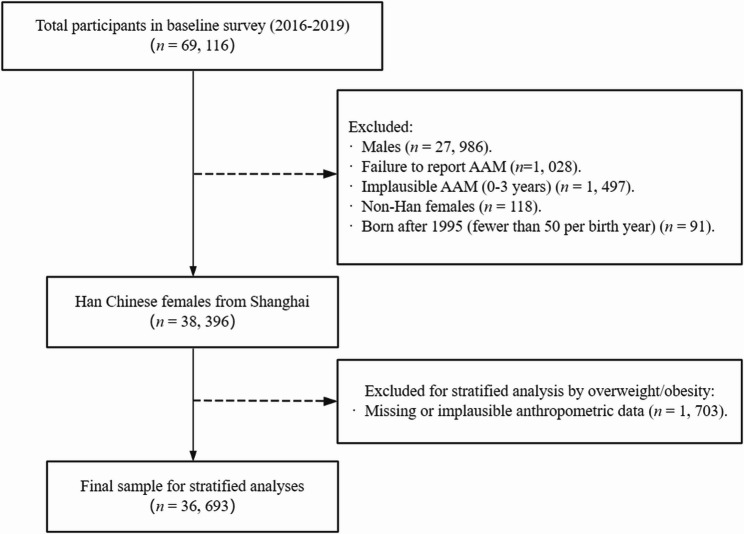
Participant Inclusion and Exclusion Flowchart

For stratified analyses by overweight/obesity status, we further excluded 1,703 individuals with missing or implausible anthropometric data (e.g., height and weight), resulting in a final sample of 36,693 participants. The detailed exclusion process is illustrated in Fig. 1.

### Measurements

This research followed the Declaration of Helsinki, approved by the Institutional Review Board of the School of Public Health (2016-04−0586-S1). Written consent was obtained from all participants. Participants were divided into 11 cohorts based on their birth years, ranging from 1942 to 1995, with the initial cohort spanning from 1942 to 1945 and subsequent cohorts defined at 5-year intervals. Within each birth-year cohort, individuals were further classified into five categories corresponding to the 10th, 25th, 50th, 75th, and 90th percentiles of AAM. The survey questionnaire included an open-ended question to ascertain AAM through self-reporting by the participants: “How old were you at menarche (when you first menstruated)?”

Body mass index (BMI) was calculated as the ratio of weight (kg) to height squared (m²). Considering the physical characteristics and disease risk data of the Chinese population, the National Health and Family Planning Commission of the People’s Republic of China recommends the adoption of lower cut-off points compared to those proposed by the World Health Organization: a BMI of ≥ 24.0 kg/m² but < 28.0 kg/m² is defined as overweight, and a BMI of ≥ 28.0 kg/m² is defined as obese [38]. Based on these criteria, participants were categorized into two groups: non-overweight/obese (NOO, < 24.0 kg/m²) and overweight/obese (OO, ≥ 24.0 kg/m²).

Sociodemographic and Anthropometric characteristics, including height, weight, overweight/obesity status, educational attainment, and whether the birth was full-term, are shown in Supplementary Table 1.

### Statistical analysis

The AAM was recorded in whole years; for instance, an age of 14 years encompassed the range from 14.00 to 14.99 years. For statistical purposes, a representative age was computed by adding 0.5 to the self-reported age, ensuring precise representation of the AAM range for each individual.

In order to evaluate the time trends of AAM as a continuous variable, quadratic regression analyses was conducted., which can better fit the accelerating or decelerating trends of AAM over a long time span. The time trends in AAM were further examined across 11 birth-year groups, each spanning a 5-year interval, using quantile regression analysis, which can supplement the limitations of mean analysis, thereby providing a more comprehensive understanding of the overall characteristics of AAM changes. Specifically, the mean AAM and its 95% confidence intervals (*CI*s) were calculated for each birth-year group. The trends across different percentile groups (10th, 25th, 50th, 75th, and 90th) were also analyzed to evaluate the variability in AAM over time. Additionally, stratified analyses were performed based on overweight/obesity status in adulthood to examine the possible relationship between percentile group AAM and adult overweight/obesity.

All statistical analyses were performed using R4.3.2 and SPSS19.0. Statistical significance was determined using a two-sided p-value threshold of < 0.05.

## Results

### Whole trend of AAM in Shanghai Han females

**Fig. 2:**
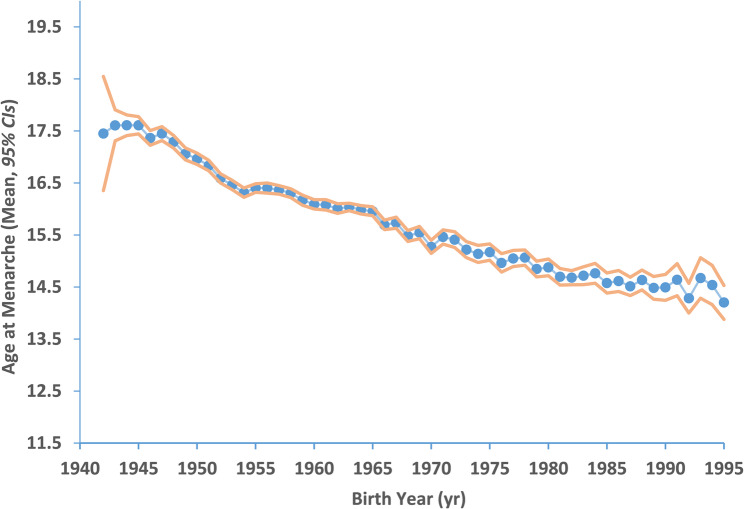
Cumulative proportion of AAM in Shanghai Han Females. Points represent the mean AAM of each birth year. The orange area represents the 95% *CI*s of the population. AAM, age at menarche; *CI*s, confidence intervals.

Overall, the mean AAM of Han females in shanghai exhibited a significant secular trend towards earlier AAM from 1942 to 1995 in the twentieth century, with extensive data revealing a decrease from 17.45 ± 2.50 years in Han females born in 1942 to 14.20 ± 1.33 years in those born in 1995, with an average decline of 0.68 years per decade (*p* < 0.001), as visually represented in Fig. 2.

### Trends in AAM across percentile groups

Analyses of the secular trend in AAM across various birth-year groups, from those born before 1945 through to the 1991–1995, reveal significant variations in the rate of decline according to the percentile group of AAM. In the 50th percentile group (median AAM), the age decreased from 17.5 to 14.5 years. Among the 10th percentile group, which represents earlier menarche, the AAM declined from 14.5 years to 12.5 years. Conversely, in the 90th percentile group, indicative of later menarche, the AAM range shifted from 20.5 to 16.5 years. These findings are summarized in Table 1.

**Table 1:** Trends in AAM across percentile groups among 38,396 Han females in Shanghai born between 1942 and 1995

The Birth-year Group	*N*	Mean ± SD	The Percentile group of AAM (years)				
			10th	25th	50th	75th	90th
1942–1945	1271	17.61 ± 2.12	14.5	16.5	**17.5**	19.5	20.5
1946–1950	5085	17.20 ± 2.00	14.5	15.5	**17.5**	18.5	19.5
1951–1955	7848	16.51 ± 1.82	14.5	15.5	**16.5**	17.5	18.5
1956–1960	7251	16.28 ± 1.75	14.5	15.5	**16.5**	17.5	18.5
1961–1965	6704	16.01 ± 1.58	14.5	15.5	**16.5**	16.5	17.5
1966–1970	3903	15.57 ± 1.54	13.5	14.5	**15.5**	16.5	17.5
1971–1975	1950	15.29 ± 1.53	13.5	14.5	**15.5**	16.5	17.5
1976–1980	1666	14.96 ± 1.47	13.5	13.5	**14.5**	15.5	16.5
1981–1985	1374	14.68 ± 1.40	13.5	13.5	**14.5**	15.5	16.5
1986–1990	974	14.55 ± 1.45	12.5	13.5	**14.5**	15.5	16.5
1991–1995	370	14.47 ± 1.47	12.5	13.5	**14.5**	15.5	16.5

*AAM *age at menarche, *SD* Standard Deviation, Total sample size: *N* = 38,396.

**Fig. 3:**
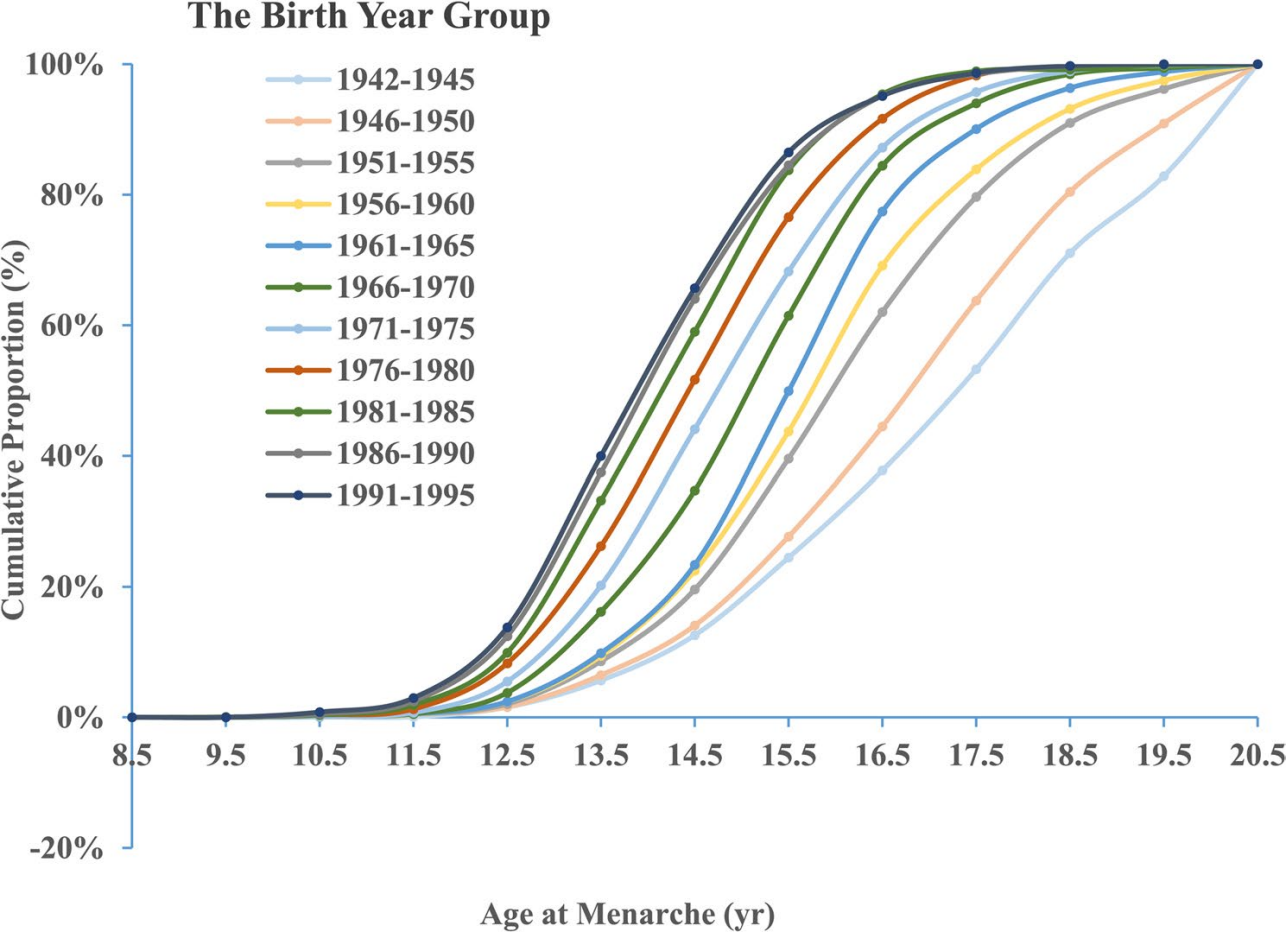
Cumulative proportion of AAM across 11 birth-year groups

The examination of the cumulative proportion of menarche across different birth-year groups indicates a secular trend towards earlier AAM. This trend is marked by a leftward shift in the distribution curves, indicative of an earlier onset of menarche in more recent birth cohorts. Specifically, the curves representing more contemporary birth-year groups, such as 1991–1995, exhibit a steeper ascent, reaching the 0.5 cumulative proportion at a significantly lower age compared to earlier cohorts, such as those born between 1942 and 1945. Additionally, the narrowing of the curves in more recent birth-year groups suggests a reduction in the variability of the AAM. The range between the 10th and 90th percentiles is notably smaller in recent birth-year groups compared to earlier ones, indicating a more homogeneous distribution of the AAM among participants, as visually represented in Fig. 3.

### Association of status AAM trends with overweight/obesity

To further investigate the association of status AAM trends with overweight/obesity, we conducted a stratified analysis based on overweight/obesity, encompassing a total sample size of 36,693 individuals. Over the period under examination, the mean AAM in the NOO group ranged from 17.68 ± 2.10 years to 14.54 ± 1.46 years, while that of the OO group decreased from 17.54 ± 2.12 years to 14.50 ± 1.55 years.

Meanwhile, the OO group showed an earlier transition to lower AAM compared to the NOO group. For instance, at the 10th percentile, the AAM reached 13.5 years in the OO group by the 1956–1960 birth-year group, compared to the 1966–1970 group in NOO girls. At the 25th percentile, the AAM was 14.5 years in the OO group by 1961–1965, which was achieved by the NOO group only in the subsequent 1966–1970 birth-year group. At the 50th percentile, the AAM was 14.5 years in the OO group by the 1976–1980 birth-year group, compared to the NOO group, whose median AAM was 15.5 years during the same period. At the 90th percentile, the AAM reached 17.5 years in the OO group by the 1961–1965 birth-year group, whereas in the NOO group, this milestone was not reached until the 1966–1970 birth-year group, as delineated in Table 2.

**Table 2:** Trends in AAM across percentile groups among 36,693 Han females in Shanghai born between 1942 and 1995, stratified by Overweight/Obesity status

Status	The Birth-year Group	*N*	Mean ± SD	The Percentile group of AAM (years)				
				10th	25th	50th	75th	90th
Overweight/Obesity (*N* = 17235)								
	1942–1945	672	17.54 ± 2.12	14.5	15.5	**17.5**	19.5	20.5
	1946–1950	2737	17.25 ± 1.98	14.5	15.5	**17.5**	18.5	19.5
	1951–1955	3986	16.51 ± 1.82	14.5	15.5	**16.5**	17.5	18.5
	1956–1960	3463	16.20 ± 1.74	13.5	15.5	**16.5**	17.5	18.5
	1961–1965	3044	15.91 ± 1.57	13.5	14.5	**15.5**	16.5	17.5
	1966–1970	1567	15.48 ± 1.51	13.5	14.5	**15.5**	16.5	17.5
	1971–1975	677	15.26 ± 1.50	13.5	14.5	**15.5**	16.5	17.5
	1976–1980	477	14.78 ± 1.55	12.5	13.5	**14.5**	15.5	16.5
	1981–1985	310	14.48 ± 1.39	12.5	13.5	**14.5**	15.5	16.5
	1986–1990	220	14.30 ± 1.43	12.5	13.5	**14.5**	15.5	16.5
	1991–1995	82	14.50 ± 1.55	12.5	13.5	**14.5**	15.5	16.5
Non-Overweight/Obesity (*N* = 19458)								
	1942–1945	557	17.68 ± 2.10	14.5	16.5	**17.5**	19.5	20.5
	1946–1950	2131	17.15 ± 2.01	14.5	15.5	**17.5**	18.5	19.5
	1951–1955	3530	16.52 ± 1.82	14.5	15.5	**16.5**	17.5	18.5
	1956–1960	3463	16.36 ± 1.76	14.5	15.5	**16.5**	17.5	18.5
	1961–1965	3406	16.10 ± 1.58	14.5	15.5	**16.5**	16.5	18.5
	1966–1970	2193	15.65 ± 1.55	13.5	14.5	**15.5**	16.5	17.5
	1971–1975	1194	15.32 ± 1.56	13.5	14.5	**15.5**	16.5	17.5
	1976–1980	1096	15.06 ± 1.44	13.5	14.5	**15.5**	16.5	16.5
	1981–1985	959	14.79 ± 1.41	13.5	13.5	**14.5**	15.5	16.5
	1986–1990	672	14.67 ± 1.47	12.5	13.5	**14.5**	15.5	16.5
	1991–1995	257	14.54 ± 1.46	12.5	13.5	**14.5**	15.5	16.5

*AAM* age at menarche, *SD* Standard Deviation.

## Variations in the AAM by percentile group and Overweight/Obesity status

Employing quantile regression analysis, we examined the relationship between percentile group AAM and adult overweight/obesity. The overall analysis revealed a decrease in the mean AAM by 0.068 years per annual increase in birth year (95% *CI*: −0.070 to −0.066). This trend varied significantly across different percentile groups of AAM: a decrease of 0.048 years per year in the 10th percentile group (95% *CI*: −0.050 to −0.045), 0.069 years per year in the 50th percentile group (95% *CI*: −0.070 to −0.067), and 0.091 years per year in the 90th percentile group (95% *CI*: −0.094 to −0.088), with all p-values < 0.001.

When stratified by overweight/obesity status, a more significant downward trend in AAM was observed in the OO group, with an annual reduction of 0.076 years (95% *CI*: −0.079 to −0.073). In contrast, the NOO group experienced an annual decrease of 0.064 years (95% *CI*: −0.066 to −0.062). Further details are provided in Table 3.

**Table 3:** The relationship of percentile group and Overweight/Obesity status on AAM

Percentile group of AAM	Total (*N* = 38396)		Non- Overweight/Obesity (*N* = 19458)		Overweight/Obesity (*N* = 17235)	
	Mean (OLS)	95% CIs	Mean (OLS)	95% CIs	Mean (OLS)	95% CIs
10th	−0.048	(−0.050, −0.045)	−0.043	(−0.048, −0.039)	−0.053	(−0.056, −0.049)
25th	−0.057	(−0.059, −0.055)	−0.056	(−0.058, −0.053)	−0.063	(−0.066, −0.059)
50th	−0.069	(−0.070, −0.067)	−0.065	(−0.067, −0.062)	−0.081	(−0.083, −0.079)
75th	−0.083	(−0.085, −0.081)	−0.077	(−0.08, −0.074)	−0.091	(−0.094, −0.088)
90th	−0.091	(−0.094, −0.088)	−0.087	(−0.092, −0.082)	−0.100	(−0.105, −0.095)
Total	−0.068	(−0.070, −0.066)	−0.064	(−0.066, −0.062)	−0.076	(−0.079, −0.073)

*AAM* age at menarche, *OLS* ordinary least squares, *CI*s Confidence Intervals.

## Discussion

This study leveraged the large-scale baseline survey data from the SSACB to investigate the secular trends in AAM among 38,396 Han females from Shanghai, China, during the second half of the twentieth century. Our analysis revealed a decline in mean AAM, from 17.45 ± 2.50 years for those born in 1942 to 14.20 ± 1.33 years for those born in 1995, with an average decrease of 0.68 years per decade (equivalent to 0.068 years per birth-year increment; *p* < 0.001). These findings highlight a substantial shift in the timing of menarche over a span of 54 years, reflecting broader changes in health and development among this population.

The trend of earlier AAM may be driven by multiple factors. From a biological perspective, genetic factors influence the timing of puberty onset by regulating key nodes of the hypothalamic-pituitary-gonadal axis, such as hypothalamic GnRH pulse secretion [8]. Additionally, environmental endocrine disruptors (e.g., Bisphenol A, phthalates) enter the body through inhalation, ingestion, or direct contact [39] and disrupt hypothalamic-pituitary-gonadal axis regulation, leading to early puberty [7, 9].

At the socioeconomic level, economic development has not only shifted diets from a subsistence-oriented model to an abundance-oriented one but also restructured the overall living environment. Overnutrition is indeed a significant risk factor for earlier menarche [25, 40], but it is not the sole contributing factor. A cohort study of women born in the UK between 1908 and 1993 showed that after a period of stabilization in the mid-20th century, menarcheal age resumed its downward trend in recent cohorts, with differences observed across socio-economic groups [26]. In the US, a cohort study of 71,341 women found that the trend toward earlier menarche was more pronounced among racial and ethnic minority groups and individuals of low SES, and this disparity could not be fully explained by BMI [27]. Prospective studies further confirm that low Socio-Economic Status (SES) accelerates puberty onset (rather than shortening its tempo), contributing to earlier menarche. This association between SES and the timing of puberty onset remains significant even after adjusting for BMI [41]. Data from Japanese women over a century revealed that key inflection points in the decline of menarcheal age coincided with major historical events such as wars and economic recovery, demonstrating that dramatic changes in the socio-economic environment can directly reshape reproductive development [42]. Beyond macro-level socio-economic factors, gene-environment interactions at the individual level also play a crucial role in regulating menarche. Obesity-related genetic loci interact with high - SES environments to significantly amplify the risk of early puberty in Chinese girls [43]. Therefore, earlier menarche is a result jointly driven by diet, lifestyle, genetics, environment, socio-economic background, historical changes, and gene-environment interactions.

Historically, the trend aligns with observations in other Asian countries and regions within China [24]– [25, 29–34]. Yet, a 2017–2019 nationwide survey found that the median AAM has stabilised at 12.39 years, indicating that the rapid decline observed in earlier decades has now plateaued [40]. However, in some Western countries, the rate of decline has slowed or stabilized [28]. The observed differences in long-term trends across regions may be attributed not only to biological and genetic factors but also to variations in ethnicity [27], socioeconomic status [26]– [27, 41–43], environmental exposures [7]– [8, 44]– [45], and dietary and lifestyle factors [46–49]. Although these factors provide a wide background for understanding the AAM’s secular trends, it is also important to consider specific historical events and their potential impact on these trends. Previous studies have shown that malnutrition during famine may lead to delayed puberty [45]. However, this study focuses on long-term AAM trends among Han women in Shanghai—a highly urbanized area less severely affected by famine than rural regions or the national average [50]. Moreover, Shanghai’s rapid economic recovery, social development, and improvements in SES and medical care may have alleviated famine’s long-term impact on AAM. These factors likely contributed to the non-significant changes in AAM observed among females born during the famine years in our study. However, considering the differences in social protection policies and the speed of nutritional recovery across regions, future studies could further explore the potential impact of famine on AAM in other populations.

Over the past few decades, Shanghai has undergone rapid urban expansion. Urbanization-driven factors (e.g., sedentary lifestyles) [51] and westernization of dietary structures [52, 53] (such as animal food consumption exceeding the recommended maximum intake by more than 30% [54]) may have exerted profound impacts on AAM [55–59]. Additionally, this study observed decreased variability in the AAM among recently born Han Chinese females in Shanghai, with a more uniform distribution compared to earlier periods. This trend is likely due to China’s sustained efforts to implement policies aimed at improving overall health, particularly by addressing undernutrition [60]. As China’s socioeconomic development has progressed, there has been a continuous improvement in the prevalence of stunting and thinness [61], and a rapid shift in the nutritional status of children and adolescents across different ethnic groups [62]. However, the advancement of urbanization has been accompanied by a decline in the physical health levels of students in highly urbanized areas [60]. Therefore, it is imperative to develop targeted regional strategies that employ a multi-sectoral approach, involving education, healthcare, and urban planning, to address the unique challenges posed by urbanization [63]. This approach should advocate for a shift from a focus on undernutrition to a more comprehensive strategy that addresses both undernutrition and overnutrition [64], thereby promoting the healthy growth of children and adolescents in highly urbanized areas, including Shanghai, in the face of evolving health needs and rapid urbanization.

A national cross-sectional health survey conducted from 2017 to 2019, involving 108,343 girls aged 3–18 years, showed that the median AAM was 12.39 years (95% CI: 12.37 to 12.42), with no significant change observed over the past decade [40]. Additionally, the prevalence of early puberty was higher in girls residing in eastern, central, and northern regions compared to other areas [40]. Against the backdrop of earlier menarche, the AAM significantly impacts total height growth during adolescence [65] and is associated with various health indicators, including self-rated health status, psychological stress, and emotions among Asian women [66], as well as mental health issues in girls [67]– [68]. In addition, early menarche seems to have an exact additional effect on all causes of mortality [69]. It can be said that the AAM has a great influence on women’s physical and mental health throughout their lives. Therefore, even though the downward trend of AAM of Shanghai women in the eastern part of China may gradually slow down or tend to be stable, consistent with the national plateau observed since 2017 [40], from the perspective of public health, the impact of earlier menarche on women’s health should be paid more attention by health management departments.

Meanwhile, the study found that the AAM of overweight/obese women in adulthood was earlier and decreased faster, emphasizing the potential relationship between AAM and adult overweight/obesity. Percentile analysis further revealed that all AAM groups showed a downward trend, with more pronounced declines observed in overweight/obese participants. Notably, the later menarche group (90th percentile) exhibited a greater decline than the earlier menarche group (10th percentile)—a pattern consistent with previous research [24]—suggesting that the later menarche group may contribute more substantially to the overall secular decline in AAM. This finding provides a basis for further study on the complex relationship between puberty initiation and adult obesity.

A large-scale national study has shown [70] that women with earlier menarche in China have a higher risk of obesity, that is, AAM was negatively correlated with obesity risk (Odds Ratio: 0.968; 95% CI: 0.961 to 0.975). However, previous studies have also found that preventing childhood obesity is the key to prevent early puberty, adult obesity and related cardiovascular risks [71]. Therefore, the complex causal relationship between earlier menarche and obesity in females requires further longitudinal studies and long-term observations to be fully elucidated.

Given the trend of earlier AAM and its potential impact on health, the findings of this study will have positive implications for optimizing policies related to children and adolescents’ health in China, as well as for advancing reproductive health education among children and adolescents. While continuously strengthening adolescent health education [72], efforts should be made to promote menstrual hygiene management in schools [73], and further explore an integrated intervention package encompassing nutrition, physical activity, and psychological support [64, 74–76]. It is essential to utilize the new complete growth reference for urban Chinese children to assess the growth and nutritional status of Chinese urban children throughout childhood [77], and to increasingly focus on young children and developmental issues [78]. Additionally, relevant administrative departments are advised to prioritize emerging adolescent health issues in China as key policy actions within the framework of Healthy China 2030 [79], sustain attention to adolescent reproductive health indicators [80], and establish regional databases on pubertal development. These measures will accelerate the realization of a new model for health management in rapidly urbanizing areas, thereby supporting the comprehensive health development of children and adolescents.

The primary strength of this study lies in its comprehensive utilization of large-scale community-based survey data from Shanghai, elucidating the continuous decline in AAM among Han Chinese females in Shanghai over more than half a century. Although previous studies have reported the trends in AAM among over 5000 women in Shanghai and its surrounding areas [35], large-scale sample data remain essential for validating long-term trends in this region. Additionally, this study further explored the potential relationship between overweight/obesity status in adulthood and these trends, which provided valuable insights for the change of AAM.

However, there are several limitations in this study. First, while a prospective investigation would be optimal, it is impractical within the adult cohort of the SSACB. Although memory bias is a potential concern in retrospective studies, the self-reported AAM is generally considered reliable due to the significance of this life event [81]. Moreover, China’s fixed nine-year compulsory education system and school entry ages provide participants with contextual cues to aid recall. For instance, if menarche occurred in the seventh grade, this can be reasonably extrapolated to an age of approximately 13 years. Therefore, we believe that the potential for recall bias in our study is minimal. Second, because the participants of SSACB are in adulthood at the baseline survey stage, it is impossible to evaluate the BMI of participants at menarche. Despite the large sample size, the cross-sectional design ruled out the possibility of establishing causality, and the potential relationship between earlier menarche and overweight/obesity in adulthood deserves further study. Third, in view of the large sample providing insights for the AAM trend of women in Shanghai, our research can be used as a reference for comparing AAM trends in different regions. Even so, this study is only aimed at Han women in Shanghai. Given the potential variations in ethnicity, environment, socioeconomic status, and lifestyle, extrapolating these findings to other demographics or countries should be approached with caution. Forth, the study leverages ecological data from a large-scale population survey, which inherently restricts our ability to assess individual-level factors such as nutrition, diet, and lifestyle that may influence AAM.

In a word, we contend that these limitations are unlikely to directly influence our findings. This study serves as a health-related alert for women born in Shanghai from 1942 to 1995. Based on the available evidence, there is a clear secular trend toward earlier AAM among Han Chinese women in Shanghai.

## Conclusion

In summary, this study provides comprehensive insights into the secular trends in the AAM among Han Chinese females in Shanghai, revealing a significant decline over the past half-century. It is suggested that earlier menarche may increase the risk of overweight/obesity in adulthood, and attention should be paid to early weight control intervention. The research results have important reference value for formulating public health policies aimed at improving the health of Han females in highly urbanized areas.

## Supplementary Information

Supplementary Material 1: The following supporting information can be downloaded at https://link.springer.com/xxx/xx. Supplementary Table 1: Sociodemographic and Anthropometric Characteristics of the Study Population.

## Data Availability

The datasets generated and analyzed during this study are available from Genming Zhao. However, due to licensing restrictions, these data cannot be made publicly accessible. Data may be shared upon reasonable request to the authors and with the permission of Genming Zhao.

## Abbreviations

AAM: Age at Menarche
SSACB: Shanghai Suburban Adult Cohort and Biobank
BMI: Body Mass Index
NOO: Non-Overweight/Obese
OO: Overweight/Obese
CI: Confidence Interval
SD: Standard Deviation
OLS: Ordinary Least Squares
SES: Socio-Economic Status

## References

[1] Cousins S. The great puberty shift. Lancet. 2024;404(10452):511–2. 10.1016/S0140-6736(24)01640-4. 39128480 10.1016/S0140-6736(24)01640-4

[2] Hargreaves D, Mates E, Menon P, et al. Strategies and interventions for healthy adolescent growth, nutrition, and development. Lancet. 2022;399(10320):198–210. 10.1016/S0140-6736(21)01593-2. 34856192 10.1016/S0140-6736(21)01593-2

[3] Karapanou O, Papadimitriou A. Determinants of menarche. Reprod Biol Endocrinol. 2010;8:115. 10.1186/1477-7827-8-115. 20920296 10.1186/1477-7827-8-115 PMC2958977

[4] Canton APM, Tinano FR, Guasti L, et al. Rare variants in the MECP2 gene in girls with central precocious puberty: a translational cohort study. Lancet Diabetes Endocrinol. 2023;11(8):545–54. 10.1016/S2213-8587(23)00131-6. 37385287 10.1016/S2213-8587(23)00131-6 PMC7615084

[5] Thiyagarajan DK, Basit H, Jeanmonod R, Physiology. Menstrual Cycle. In: StatPearls [Internet]. Treasure Island (FL): StatPearls Publishing; 2024 Jan–. https://www.ncbi.nlm.nih.gov/books/NBK557787/29763196

[6] Zhou F, Mao J, Jin Z, Zhu L, Li X. Multi-omic analysis of precocious puberty girls: pathway changes and metabolite validation. Front Endocrinol (Lausanne). 2024;15:1285666. 10.3389/fendo.2024.1285666. 38487340 10.3389/fendo.2024.1285666 PMC10937432

[7] Lu M, Feng R, Qin Y, Deng H, Lian B, Yin C, Xiao Y. Identifying environmental endocrine disruptors associated with the age at menarche by integrating a Transcriptome-Wide association study with Chemical-Gene-Interaction analysis. Front Endocrinol (Lausanne). 2022;13:836527. 10.3389/fendo.2022.836527. 35282430 10.3389/fendo.2022.836527 PMC8907571

[8] Faienza MF, Urbano F, Moscogiuri LA, Chiarito M, De Santis S, Giordano P. Genetic, epigenetic and enviromental influencing factors on the regulation of precocious and delayed puberty. Front Endocrinol (Lausanne). 2022;13:1019468. 10.3389/fendo.2022.1019468. 36619551 10.3389/fendo.2022.1019468 PMC9813382

[9] Fudvoye J, Lopez-Rodriguez D, Franssen D, Parent AS. Endocrine disrupters and possible contribution to pubertal changes. Best Pract Res Clin Endocrinol Metab. 2019;33(3):101300. 10.1016/j.beem.2019.101300. 31401055 10.1016/j.beem.2019.101300

[10] Tzounakou AM, Stathori G, Paltoglou G, Valsamakis G, Mastorakos G, Vlahos NF, Charmandari E. Childhood Obesity, hypothalamic Inflammation, and the onset of puberty: A narrative review. Nutrients. 2024;16(11):1720. 10.3390/nu16111720. 38892653 10.3390/nu16111720 PMC11175006

[11] Leone T, Brown LJ. Timing and determinants of age at menarche in low-income and middle-income countries. BMJ Glob Health. 2020;5(12):e003689. 10.1136/bmjgh-2020-003689. 33298469 10.1136/bmjgh-2020-003689 PMC7733094

[12] Okoth K, Chandan JS, Marshall T et al. Association between the reproductive health of young women and cardiovascular disease in later life: umbrella review. BMJ. 2020;371:m3502. Published 2020 Oct 7. 10.1136/bmj.m3502 10.1136/bmj.m3502 PMC7537472 33028606

[13] Hu ZB, Lu ZX, Zhu F. Age at menarche, age at menopause, reproductive years and risk of fatal stroke occurrence among Chinese women: the Guangzhou biobank cohort study. BMC Womens Health. 2021;21(1):433. 10.1186/s12905-021-01579-9. Published 2021 Dec 28. 34961507 10.1186/s12905-021-01579-9 PMC8714414

[14] Kim C, Catov J, Schreiner PJ, et al. Women’s reproductive milestones and cardiovascular disease risk: A review of reports and opportunities from the CARDIA study. J Am Heart Assoc. 2023;12(5):e028132. 10.1161/JAHA.122.028132. 36847077 10.1161/JAHA.122.028132 PMC10111436

[15] Magnus MC, Guyatt AL, Lawn RB, et al. Identifying potential causal effects of age at menarche: a Mendelian randomization phenome-wide association study. BMC Med. 2020;18(1):71. 10.1186/s12916-020-01515-y. 32200763 10.1186/s12916-020-01515-y PMC7087394

[16] Chiou JS, Lin YJ, Chang CY, et al. Menarche—a journey into womanhood: age at menarche and health-related outcomes in East Asians. Hum Reprod. 2024;39(6):1336–50. 10.1093/humrep/deae060. 38527428 10.1093/humrep/deae060

[17] SadrAzar A, Sanaie S, Tutunchi H, Sheikh B, Faramarzi E, Jourabchi-Ghadim N. Is early age at menarche associated with multimorbidity? Findings from the Azar cohort study. Eur J Obstet Gynecol Reprod Biol. 2023;287:46–51. 10.1016/j.ejogrb.2023.05.029. 37290234 10.1016/j.ejogrb.2023.05.029

[18] Rahimi Z, Saki N, Cheraghian B, et al. Association between age at menarche and metabolic syndrome in Southwest iran: A Population-Based Case-Control study. J Res Health Sci. 2022;22(3):e00558. 10.34172/jrhs.2022.93. 36511376 10.34172/jrhs.2022.93 PMC10422154

[19] Fuhrman BJ, Moore SC, Byrne C, et al. Association of the age at menarche with Site-Specific cancer risks in pooled data from nine cohorts. Cancer Res. 2021;81(8):2246–55. 10.1158/0008-5472. 33820799 10.1158/0008-5472.CAN-19-3093 PMC8137527

[20] Harris AR, Wang T, Heng YJ, et al. Association of early menarche with breast tumor molecular features and recurrence. Breast Cancer Res. 2024;26(1):102. 10.1186/s13058-024-01839-0. 38886818 10.1186/s13058-024-01839-0 PMC11181557

[21] Jiang L, Hao Y, Wang Y, et al. Is early menarche related to depression? A meta-analysis. J Affect Disord. 2025;369:508–15. 10.1016/j.jad.2024.10.036. 39393462 10.1016/j.jad.2024.10.036

[22] Worthman CM, Trang K. Dynamics of body time, social time and life history at adolescence. Nature. 2018;554(7693):451–7. 10.1038/nature25750. 29469099 10.1038/nature25750

[23] Petersohn I, Zarate-Ortiz AG, Cepeda-Lopez AC, Melse-Boonstra A. Time trends in age at menarche and related Non-Communicable disease risk during the 20th century in Mexico. Nutrients. 2019;11(2):394. 10.3390/nu11020394. 30781889 10.3390/nu11020394 PMC6412794

[24] Lee DH, Kim J, Kim HY. Temporal trend of age at menarche in Korean females born between 1927 and 2004: a population-based study. Front Endocrinol (Lausanne). 2024;15:1399984. 10.3389/fendo.2024.1399984. 38894747 10.3389/fendo.2024.1399984 PMC11182987

[25] Ma N, Shi D, Dang JJ, et al. Secular trends and urban-rural disparities in the median age at menarche among Chinese Han girls from 1985 to 2019. World J Pediatr. 2023;19(12):1162–8. 10.1007/s12519-023-00723-9. 37093553 10.1007/s12519-023-00723-9

[26] Morris DH, Jones ME, Schoemaker MJ, Ashworth A, Swerdlow AJ. Secular trends in age at menarche in women in the UK born 1908-93: results from the breakthrough generations study. Paediatr Perinat Epidemiol. 2011;25(4):394–400. 10.1111/j.1365-3016.2011.01202.x. 21649682 10.1111/j.1365-3016.2011.01202.x

[27] Wang Z, Asokan G, Onnela JP, et al. Menarche and time to cycle regularity among individuals born between 1950 and 2005 in the US. JAMA Netw Open. 2024;7(5):e2412854. 10.1001/jamanetworkopen.2024.12854. 38809557 10.1001/jamanetworkopen.2024.12854 PMC11137638

[28] Nabhan AF, Mburu G, Elshafeey F, et al. Women’s reproductive span: a systematic scoping review. Hum Reprod Open. 2022;2022(2):hoac005. 10.1093/hropen/hoac005. 35280216 10.1093/hropen/hoac005 PMC8907405

[29] Lyu Y, Mirea L, Yang J, Warre R, Zhang J, Lee SK, Li Z. Secular trends in age at menarche among women born between 1955 and 1985 in southeastern China. BMC Womens Health. 2014;14:155. 10.1186/s12905-014-0155-0. 25495097 10.1186/s12905-014-0155-0 PMC4275952

[30] Song Y, Ma J, Agardh A, Lau PW, Hu P, Zhang B. Secular trends in age at menarche among Chinese girls from 24 ethnic minorities, 1985 to 2010. Glob Health Action. 2015;8:26929. 10.3402/gha.v8.26929. 26220757 10.3402/gha.v8.26929 PMC4518164

[31] Liu W, Yan X, Li C, et al. A secular trend in age at menarche in Yunnan Province, china: a multiethnic population study of 1,275,000 women. BMC Public Health. 2021;21(1):1890. 10.1186/s12889-021-11951-x. 34666747 10.1186/s12889-021-11951-x PMC8524999

[32] Deng Y, Liang J, Zong Y, et al. Timing of spermarche and menarche among urban students in Guangzhou, china: trends from 2005 to 2012 and association with obesity. Sci Rep. 2018;8(1):263. 10.1038/s41598-017-18423-6. 29321542 10.1038/s41598-017-18423-6 PMC5762656

[33] Hu J, Han W, Zhou M et al. Secular trends in the median age at menarche and spermarche among Chinese children from 2000 to 2019 and analysis of physical examination indicators factor. Am J Hum Biol 2024 Dec 9:e24198. 10.1002/ajhb.24198 10.1002/ajhb.24198 39653583

[34] Wang Z, Dang S, Xing Y, Li Q, Yan H. Correlation of body mass index levels with menarche in adolescent girls in Shaanxi, china: a cross-sectional study. BMC Womens Health. 2016;16:61. 10.1186/s12905-016-0340-4. 27599475 10.1186/s12905-016-0340-4 PMC5013571

[35] Jin F, Shao H, Tao M. Trends of menarcheal age in women in the reproductive age and menopause in Shanghai. Gynecol Obstet Invest. 2013;76(4):228–32. 10.1159/000355695. 24192266 10.1159/000355695

[36] GBD2021ABMIC. Global, regional, and National prevalence of adult overweight and obesity, 1990–2021, with forecasts to 2050: a forecasting study for the global burden of disease study 2021. Lancet (London England). 2025;405(10481):813–38. 10.1016/S0140-6736(25)00355-1. 10.1016/S0140-6736(25)00355-1 PMC11920007 40049186

[37] Zhao Q, Chen B, Wang R, et al. Cohort profile: protocol and baseline survey for the Shanghai suburban adult cohort and biobank (SSACB) study. BMJ Open. 2020;10:e035430. 10.1136/bmjopen-2019-035430. 32641326 10.1136/bmjopen-2019-035430 PMC7348462

[38] National Health and Family Planning Commission of. People’s Republic of China. WS/T 428–2013 Criteria of weight for adults [S]. 2013.

[39] Calcaterra V, Cena H, Loperfido F, et al. Evaluating phthalates and bisphenol in foods: risks for precocious puberty and Early-Onset obesity. Nutrients. 2024;16(16):2732. 10.3390/nu16162732. Published 2024 Aug 16. 39203868 10.3390/nu16162732 PMC11357315

[40] Liang X, Huang K, Dong G, et al. Current pubertal development in Chinese children and the impact of Overnutrition, Lifestyle, and perinatal factors. J Clin Endocrinol Metab. 2023;108(9):2282–9. 10.1012/clinem/dgad102. 36881937 10.1210/clinem/dgad102

[41] Hiatt RA, Stewart SL, Deardorff J, et al. Childhood socioeconomic status and menarche: A prospective study. J Adolesc Health. 2021;69(1):33–40. 10.1016/j.jadohealth.2021.02.003. 34172141 10.1016/j.jadohealth.2021.02.003 PMC8243506

[42] Iwase M, Taniyama Y, Koyanagi YN, et al. A century of change: unraveling the impact of Socioeconomic/Historical milestones on age at menarche and other female reproductive factors in Japan. J Epidemiol. 2024;34(8):387–92. 10.2188/jea.JE20230155. 38191181 10.2188/jea.JE20230155 PMC11230879

[43] Xue P, Lin J, Tang J, et al. Association of obesity and menarche SNPs and interaction with environmental factors on precocious puberty. Pediatr Res. 2024;96(4):1076–83. 10.1038/s41390-024-03168-6. 38649724 10.1038/s41390-024-03168-6

[44] Nyati LH, Norris SA, Micklesfield LK, et al. Growth in infancy and childhood and age at menarche in five Low- or Middle-Income countries: consortium of health orientated research in transitional societies (COHORTS). J Nutr. 2023;153(9):2736–43. 10.1016/j.tjnut.2023.07.003. 37451558 10.1016/j.tjnut.2023.07.003 PMC10517227

[45] Wu X, Bao L, Du Z, et al. Secular trends of age at menarche and the effect of famine exposure on age at menarche in rural Chinese women. Ann Hum Biol. 2022;49(1):35–40. 10.1080/03014460.2022.2041092. 35139699 10.1080/03014460.2022.2041092

[46] Xu Y, Xiong J, Wang X, He F, Cheng G. Dietary protein sources, gut microbiome, and puberty timing in children: findings from a cohort study. Signal Transduct Target Ther. 2024;9(1):167. 10.1038/s41392-024-01890-5. 38945962 10.1038/s41392-024-01890-5 PMC11214940

[47] Zhou S, Xu Y, Xiong J, Cheng G. Cross-trait multivariate GWAS confirms health implications of pubertal timing. Nat Commun. 2025;16(1):799. 10.1038/s41467-025-56191-4. 39824883 10.1038/s41467-025-56191-4 PMC11742396

[48] Iron-Segev S, Namimi-Halevi C, Dor C, et al. Early menarche is associated with disordered eating-results from a National youth survey. Pediatr Res Published Online January. 2025;16. 10.1038/s41390-025-03852-1. 10.1038/s41390-025-03852-1 PMC12507651 39821134

[49] Sumedha SS, Pathak PK. Intergenerational transitions in age at menarche: insights from Chandauli district, Uttar Pradesh, India. BMC Womens Health. 2025;25(1):9. 10.1186/s12905-024-03462-9. 39773444 10.1186/s12905-024-03462-9 PMC11705782

[50] Xu Y, Yi Q, Shan S, et al. Chinese famine exposure in early life and metabolic obesity phenotype in middle age: results from the China health and retirement longitudinal study. Front Endocrinol (Lausanne). 2022;13:975824. 10.3389/fendo.2022.975824. Published 2022 Sep 20. 36204102 10.3389/fendo.2022.975824 PMC9531307

[51] Cacciatore S, Mao S, Nuñez MV, et al. Urban health inequities and healthy longevity: traditional and emerging risk factors across the cities and policy implications. Aging Clin Exp Res. 2025;37(1):143. 10.1007/s40520-025-03052-1. Published 2025 May 7. 40332678 10.1007/s40520-025-03052-1 PMC12058932

[52] Howard AG, Attard SM, Herring AH, Wang H, Du S, Gordon-Larsen P. Socioeconomic gradients in the westernization of diet in China over 20 years. SSM Popul Health. 2021;16:100943. 10.1016/j.ssmph.2021.100943. 34703875 10.1016/j.ssmph.2021.100943 PMC8526760

[53] Adolph TE, Tilg H. Western diets and chronic diseases. Nat Med. 2024;30(8):2133–47. 10.1038/s41591-024-03165-6. 39085420 10.1038/s41591-024-03165-6

[54] Lian Y, Gu L, Yang L, Wang L, Li H. The reasonableness and Spatial differences of the food consumption structure of urban and rural residents in China, 2015–2021. Foods. 2023;12:1997. 10.3390/foods12101997. 37238815 10.3390/foods12101997 PMC10217769

[55] Pang B, Wang Q, Yang M et al. Identification and Optimization of Contributing Factors for Precocious Puberty by Machine/Deep Learning Methods in Chinese Girls. Front Endocrinol (Lausanne). 2022;13:892005. Published 2022 Jun 30. 10.3389/fendo.2022.892005 10.3389/fendo.2022.892005 PMC9279618 35846287

[56] Calthorpe L, Brage S, Ong KK. Systematic review and meta-analysis of the association between childhood physical activity and age at menarche. Acta Paediatr. 2019;108(6):1008–15. 10.1111/apa.14711. 30588652 10.1111/apa.14711 PMC6563453

[57] Nguyen NTK, Fan HY, Tsai MC, et al. Nutrient intake through childhood and early menarche onset in girls: systematic review and Meta-Analysis. Nutrients. 2020;12(9):2544. 10.3390/nu12092544. Published 2020 Aug 22. 32842616 10.3390/nu12092544 PMC7551779

[58] Gu Q, Wu Y, Feng Z, et al. Dietary pattern and precocious puberty risk in Chinese girls: a case-control study. Nutr J. 2024;23(1):14. 10.1186/s12937-024-00916-6. Published 2024 Jan 31. 38291391 10.1186/s12937-024-00916-6 PMC10829199

[59] Wu N, Ning K, Liu Y, Wang Q, Li N, Zhang L. Relationship between high-fat diet, gut microbiota, and precocious puberty: mechanisms and implications. Front Microbiol. 2025;16:1564902. 10.3389/fmicb.2025.1564902. Published 2025 Jun 4. 40535011 10.3389/fmicb.2025.1564902 PMC12174423

[60] Dong Y, Lau PWC, Dong B, et al. Trends in physical fitness, growth, and nutritional status of Chinese children and adolescents: a retrospective analysis of 1.5 million students from six successive National surveys between 1985 and 2014. Lancet Child Adolesc Health. 2019;3(12):871–80. 10.1016/S2352-4642(19)30302-5. 31582349 10.1016/S2352-4642(19)30302-5

[61] Dong Y, Jan C, Ma Y, et al. Economic development and the nutritional status of Chinese school-aged children and adolescents from 1995 to 2014: an analysis of five successive National surveys. Lancet Diabetes Endocrinol. 2019;7(4):288–99. 10.1016/S2213-8587(19)30075-0. 30902266 10.1016/S2213-8587(19)30075-0

[62] Dong Y, Ma Y, Hu P, et al. Ethnicity, socioeconomic status, and the nutritional status of Chinese children and adolescents: findings from three consecutive National surveys between 2005 and 2014. Pediatr Obes. 2020;15(11):e12664. 10.1111/ijpo.12664. 32543108 10.1111/ijpo.12664

[63] Luo D, Ma N, Liu Y, et al. Long-term trends and urban-rural disparities in the physical growth of children and adolescents in china: an analysis of five National school surveys over three decades. Lancet Child Adolesc Health. 2023;7(11):762–72. 10.1016/S2352-4642(23)00175-X. 37714171 10.1016/S2352-4642(23)00175-X

[64] Chen TJ, Dong B, Dong Y, et al. Matching actions to needs: shifting policy responses to the changing health needs of Chinese children and adolescents. Lancet. 2024;403(10438):1808–20. 10.1016/S0140-6736(23)02894-5. 38643776 10.1016/S0140-6736(23)02894-5

[65] Jo EJ, Han S, Wang K. Estimation of causal effect of age at menarche on pubertal height growth using Mendelian randomization. Genes (Basel). 2022;13(4):710. 10.3390/genes13040710. 35456516 10.3390/genes13040710 PMC9029282

[66] Yu EJ, Choe SA, Yun JW, Son M. Association of early menarche with adolescent health in the setting of rapidly decreasing age at menarche. J Pediatr Adolesc Gynecol. 2020;33(3):264–70. 10.1016/j.jpag.2019.12.006. 31874313 10.1016/j.jpag.2019.12.006

[67] Niu L, Sheffield P, Li Y. Pubertal timing, neighborhood income, and mental health in boys and girls: findings from the adolescent brain cognitive development study. Soc Sci Med. 2023;334:116220. 10.1016/j.socscimed.2023.116220. 37690156 10.1016/j.socscimed.2023.116220

[68] Kretzer S, Lawrence AJ, Pollard R, et al. The dynamic interplay between puberty and structural brain development as a predictor of mental health difficulties in adolescence: A systematic review. Biol Psychiatry. 2024;96(7):585–603. 10.1016/j.biopsych.2024.06.012. 38925264 10.1016/j.biopsych.2024.06.012 PMC11794195

[69] Zhang X, Liu L, Song F, Song Y, Dai H. Ages at menarche and menopause, and mortality among postmenopausal women. Maturitas. 2019;130:50–6. 10.1016/j.maturitas.2019.10.009. 31706436 10.1016/j.maturitas.2019.10.009

[70] Chen L, Huang Y, Zheng C, Wang X, Zhang L, Cao X, Cai J, Hu Z, Tian Y, Gu R, Wang Z. Relation of reproductive lifespan with obesity in Chinese women: results from a large representative nationwide population. J Womens Health (Larchmt). 2025;34(3):e392–400. 10.1089/jwh.2023.0917. Epub 2024 Dec 9. PMID: 39648766. 39648766 10.1089/jwh.2023.0917

[71] O’Keeffe LM, Frysz M, Bell JA, Howe LD, Fraser A. Puberty timing and adiposity change across childhood and adolescence: disentangling cause and consequence. Hum Reprod. 2020;35(12):2784–92. 10.1093/humrep/deaa213. 33242326 10.1093/humrep/deaa213 PMC7744159

[72] Ma X, Yang Y, Chow KM, Zang Y. Chinese adolescents’ sexual and reproductive health education: A quasi-experimental study. Public Health Nurs. 2022;39(1):116–25. 10.1111/phn.12914. 33949703 10.1111/phn.12914 PMC9292843

[73] Sommer M, Caruso BA, Torondel B et al. Menstrual hygiene management in schools: midway progress update on the MHM in Ten 2014–2024 global agenda. Health Res Policy Syst. 2021;19(1):1. Published 2021 Jan 2. 10.1186/s12961-020-00669-8 10.1186/s12961-020-00669-8 PMC7776301 33388085

[74] Chen P, Wang D, Shen H, et al. Physical activity and health in Chinese children and adolescents: expert consensus statement (2020). Br J Sports Med. 2020;54(22):1321–31. 10.1136/bjsports-2020-102261. 32471813 10.1136/bjsports-2020-102261 PMC7606574

[75] Cai S, Wang H, Zhang YH, et al. Could physical activity promote indicators of physical and psychological health among children and adolescents? An umbrella review of meta-analyses of randomized controlled trials. World J Pediatr. 2025;21(2):159–73. 10.1007/s12519-024-00874-3. 39847308 10.1007/s12519-024-00874-3

[76] Luo D, Dashti SG, Sawyer SM, Vijayakumar N. Pubertal hormones and mental health problems in children and adolescents: a systematic review of population-based studies. EClinicalMedicine. 2024;76:102828. Published 2024 Oct 1. 10.1016/j.eclinm.2024.102828 10.1016/j.eclinm.2024.102828 PMC11472636 39403116

[77] Wu W, Chen J, Mo M, et al. Construction of a new complete growth reference for urban Chinese children. BMC Public Health. 2022;22(1):2345. 10.1186/s12889-022-14702-8. Published 2022 Dec 14. 36517789 10.1186/s12889-022-14702-8 PMC9749202

[78] Qu D, Wen X, Cheng X, et al. School mental health prevention and intervention strategies in china: a scoping review. Lancet Reg Health West Pac. 2024;53:101243. 10.1016/j.lanwpc.2024.101243. Published 2024 Nov 21. 39641001 10.1016/j.lanwpc.2024.101243 PMC11617956

[79] Dong B, Zou Z, Song Y, et al. Adolescent health and healthy China 2030: A review. J Adolesc Health. 2020;67(5S):S24–31. 10.1016/j.jadohealth.2020.07.023. 33246530 10.1016/j.jadohealth.2020.07.023

[80] Xu R, Song Y, Hu P, et al. Towards comprehensive National surveillance for adolescent health in china: priority indicators and current data gaps. J Adolesc Health. 2020;67(5S):S14–23. 10.1016/j.jadohealth.2020.05.043. 33246529 10.1016/j.jadohealth.2020.05.043

[81] Lundblad MW, Jacobsen BK. The reproducibility of self-reported age at menarche: the Tromsø study. BMC Womens Health. 2017;17(1):62. 10.1186/s12905-017-0420-0. 28830397 10.1186/s12905-017-0420-0 PMC5568259

