# Supplementary material for: Secular trends in age at menarche among Han Chinese females in Shanghai: a large-scale community-based study
Source: BMC Womens Health. 2025 Nov 3;25:533. doi: 10.1186/s12905-025-04064-9 (PMC12581527; doi:10.1186/s12905-025-04064-9)
Supplement: Supplementary file 1 — Supplementary Material 1: The following supporting information can be downloaded at https://link.springer.com/xxx/xx. Supplementary Table 1: Sociodemographic and Anthropometric Characteristics of the Study Population. [file 12905_2025_4064_MOESM1_ESM.docx]

**Secular Trends in Age at Menarche among Han Chinese Females in Shanghai: A Large-Scale Community-Based Study**

**Xiaoli Xu ^1^, Genming Zhao ^2^, Xing Liu** **^2^, Na Wang ^2^, Xiaohua Liu ^1^, Yonggen Jiang ^3^, Qian Peng ^4^, Jianhua Shi ^5^, Yuping Cheng ^1^ , Mengru He ^1^, Dandan He ^1,*^, Huilin Xu ^1,*^**

1 Minhang District Center for Disease Control and Prevention (Minhang District Institute of Health Supervision), Shanghai 201101, China; lisa861227@126.com (X.X.);

2 School of Public Health, Fudan University, Shanghai 200032, China;

3 Songjiang District Center for Disease Control and Prevention (Songjiang District Institute of Health Supervision), Shanghai 201600, China;

4 Jiading District Center for Disease Control and Prevention (Jiading District Institute of Health Supervision), Shanghai 201800, China;

5 Xuhui District Center for Disease Control and Prevention (Xuhui District Institute of Health Supervision), Shanghai 200237, China;

* Correspondence: dandae9@163.com (D.H.); iamxuhuilin@163.com (H.X.);

**Supplementary Table 1.** Sociodemographic and Anthropometric Characteristics of the Study Population

| **Characteristic** | **Total** | **The Birth-year Group** | | | | | | | | | | |
| --- | --- | --- | --- | --- | --- | --- | --- | --- | --- | --- | --- | --- |
|  |  | **1942-**  **1945** | **1946-**  **1950** | **1951-**  **1955** | **1956-**  **1960** | **1961-**  **1965** | **1966-**  **1970** | **1971-**  **1975** | **1976-**  **1980** | **1981-**  **1985** | **1986-**  **1990** | **1991-**  **1995** |
| **Number of individuals** | 38396 | 1271 | 5085 | 7848 | 7251 | 6704 | 3903 | 1950 | 1666 | 1374 | 974 | 370 |
| Age at baseline (years old) | 58 (51, 65) | 73 (72, 73) | 69 (68, 70) | 64 (63, 66) | 59 (58, 60) | 54 (53, 55) | 50 (48, 51) | 44 (43, 46) | 39 (38, 40) | 35 (33, 36) | 29 (28, 30) | 25 (23, 26) |
| Educational attainment |  |  |  |  |  |  |  |  |  |  |  |  |
| Primary school or below | 14971 (38.99) | 1004 (78.99) | 3285 (64.60) | 4837 (61.63) | 2918 (40.24) | 1402 (20.91) | 1070 (27.42) | 291 (14.92) | 117 (7.02) | 30 (2.18) | 12 (1.23) | 5 (1.35) |
| Junior high school | 13883 (36.16) | 174 (13.69) | 1047 (20.59) | 2136 (27.22) | 2420 (33.38) | 3948 (58.89) | 2254 (57.75) | 1014 (52) | 562 (33.73) | 215 (15.65) | 91 (9.34) | 22 (5.95) |
| Senior high school or above | 9542 (24.85) | 93 (7.32) | 753 (14.81) | 875 (11.15) | 1913 (26.38) | 1354 (20.20) | 579 (14.83) | 645 (33.08) | 987 (59.25) | 1129 (82.17) | 871 (89.43) | 343 (92.70) |
| Full-term birth |  |  |  |  |  |  |  |  |  |  |  |  |
| Yes | 27119 (70.63) | 820 (64.52) | 3448 (67.81) | 5248 (66.87) | 4934 (68.05) | 4675 (69.74) | 2799 (71.71) | 1519 (77.90) | 1384 (83.07) | 1161 (84.50) | 813 (83.47) | 318 (85.95) |
| No | 1189 (3.10) | 31 (2.44) | 133 (2.61) | 258 (3.29) | 215 (2.96) | 196 (2.92) | 101 (2.59) | 55 (2.82) | 53 (3.18) | 75 (5.46) | 52 (5.34) | 20 (5.40) |
| Unknown | 10088 (26.27) | 420 (33.04) | 1504 (29.58) | 2342 (29.84) | 2102 (28.99) | 1833 (27.34) | 1003 (25.70) | 376 (19.28) | 229 (13.75) | 138 (10.04) | 109 (11.19) | 32 (8.65) |
|  |  |  |  |  |  |  |  |  |  |  |  |  |
| **Remaining individuals*** | 36693 | 1229 | 4868 | 7516 | 6926 | 6450 | 3760 | 1871 | 1573 | 1269 | 892 | 339 |
| Height* (cm) | 156.50 (152.50, 160.20) | 151.00 (147.42, 155.00) | 153.33 (149.50, 157.00) | 155.00 (151.50, 159.00) | 156.00 (152.20, 160.00) | 157.50 (154.00, 161.00) | 158.00 (154.90, 162.00) | 158.00 (154.70, 162.00) | 159.00 (156.00, 163.00) | 160.00 (156.18, 163.50) | 160.20 (157.00, 164.40) | 161.00 (157.00, 165.00) |
| Weight* (kg) | 58.00 (52.80, 64.00) | 55.50 (50.33, 62.23) | 58.00 (52.00, 64.00) | 58.40 (53.00, 64.20) | 58.40 (53.20, 64.20) | 59.00 (54.00, 64.90) | 58.70 (53.31, 64.20) | 57.67 (52.60, 63.00) | 57.00 (52.00, 62.23) | 55.80 （51.00， 61.80） | 55.05 (49.90, 62.65) | 54.50 (48.50, 63.00) |
| BMI* (kg/m2) | 23.76 (21.73, 26.06) | 24.49 (22.31, 27.18) | 24.54 (22.31, 26.92) | 24.24 (22.23, 26.48) | 24.00 (22.06, 26.10) | 23.80 (21.87, 25.95) | 23.42 (21.63, 25.59) | 22.97 (21.20, 24.97) | 22.30 (20.54, 24.61) | 21.60 (20.02, 23.93) | 21.50 (19.57, 23.96) | 20.81 (19.07, 23.83) |
| Overweight/obesity status* |  |  |  |  |  |  |  |  |  |  |  |  |
| Yes | 17235 (46.97) | 672 (54.68) | 2737 (56.22) | 3986 (53.03) | 3463 (50.00) | 3044 (47.19) | 1567 (41.68) | 677 (36.18) | 477 (30.32) | 310 (24.43) | 220 (24.66) | 82 (24.19) |
| No | 19458 (53.03) | 557 (45.32) | 2131 (43.78) | 3530 (46.97) | 3463 (50.00) | 3406 (52.81) | 2193 (58.32) | 1194 (63.82) | 1096 (69.68) | 959 (75.57) | 672 (75.34) | 257 (75.81) |
| The data was displayed in terms of frequency (%), mean ± SD or median (*IQR*). * 1,703 individuals with missing or implausible anthropometric data. | | | | | | | | | | | | |
